# Psychosocial and environmental determinants of child cognitive development in rural south africa and tanzania: findings from the mal-ed cohort

**DOI:** 10.1186/s12889-020-08598-5

**Published:** 2020-04-16

**Authors:** Fabrizio Drago, Rebecca J. Scharf, Angelina Maphula, Emanuel Nyathi, Tjale C. Mahopo, Erling Svensen, Estomih Mduma, Pascal Bessong, Elizabeth T. Rogawski McQuade

**Affiliations:** 1 University of Virginia School of Medicine, Cardiovascular Research Center, 415 Lane Rd (MR5), Room: G231, PO Box 801394, Charlottesville, VA 22908 USA; 2 Department of Pediatrics, University of Virginia, Charlottesville, USA; 3 Department of Psychology, University of Venda, Thohoyandou, South Africa; 4 Department of Animal Science, University of Venda, Thohoyandou, South Africa; 5 Department of Nutrition, University of Venda, Thohoyandou, South Africa; 6 Centre for International Health, University of Bergen, Bergen, Norway; 7 Haydom Global Health Research Centre, Haydom, Tanzania; 8 Department of Microbiology, University of Venda, Thohoyandou, South Africa; 9 Department of Public Health Sciences and Division of Infectious Diseases & International Health, University of Virginia, Charlottesville, USA

**Keywords:** Child Development, Developing Countries, Community Health, Epidemiology

## Abstract

**Background:**

Approximately 66% of children under the age of 5 in Sub-Saharan African countries do not reach their full cognitive potential, the highest percentage in the world. Because the majority of studies investigating child cognitive development have been conducted in high-income countries (HICs), there is limited knowledge regarding the determinants of child development in low- and middle-income countries (LMICs).

**Methods:**

This analysis includes 401 mother-child dyads from the South Africa and Tanzania sites of the Etiology, Risk Factors, and Interactions of Enteric Infections and Malnutrition and the Consequences for Child Health and Development (MAL-ED) longitudinal birth cohort study. We investigated the effect of psychosocial and environmental determinants on child cognitive development measured by the Wechsler Preschool Primary Scales of Intelligence (WPPSI) at 5 years of age using multivariable linear regression.

**Results:**

Socioeconomic status was most strongly associated with child cognitive development (WPSSI Score Difference (SD):14.27, 95% CI:1.96, 26.59). Modest associations between the organization of the home environment and its opportunities for cognitive stimulation and child cognitive development were also found (SD: 3.08, 95% CI: 0.65, 5.52 and SD: 3.18, 95% CI: 0.59, 5.76, respectively).

**Conclusion:**

This study shows a stronger association with child cognitive development at 5 years of age for socioeconomic status compared to more proximal measures of psychosocial and environmental determinants. A better understanding of the role of these factors is needed to inform interventions aiming to alleviate the burden of compromised cognitive development for children in LMICs.

## Background

Approximately 66% of children under the age of 5 in Sub-Saharan African countries do not reach their full cognitive potential, the highest percentage in the world [1]. Children who do not fully develop to the level of cognitive development that would be expected in an optimal environment are less likely to enroll in and complete primary school [2–4]. These educational disadvantages can have lasting effects and are associated with adverse outcomes in adult life, e.g., lower incomes, high fertility rates, and suboptimal care for their own children [2, 5]. Because of its lifelong ramifications, delayed cognitive development contributes to intergenerational transmission of poverty and can thus have broader consequences for the economic development of low- and middle-income countries (LMICs) [2].

Children’s cognitive development is affected by several types of factors including: (1) biological (e.g., child birth weight, nutrition, and infectious diseases) [6, 7], (2) socio-economic (e.g., parental assets, income, and education) [8], (3) environmental (e.g., home environment, provision of appropriate play material, and access to healthcare) [6], and (4) psychosocial (e.g., parental mental health, parent-child interactions, cognitive stimulation, and learning opportunities) [9–11]. Household environments are the context within which a significant part of children’s development occurs. Studies show that there is a positive association between a nurturing home and optimal learning environment and children’s health and development [12–14].

Trials among children exposed to adverse household conditions have shown that early childhood parenting interventions can improve children’s cognitive development, educational achievements, and mental health outcomes [15]. Similar studies also have shown that cognitive development is associated with adult wage earning and financial growth in the subsequent generation [3, 16]. For example, a study conducted in Uganda by Singla et al. showed that children of parents who were given a parenting intervention presented higher cognitive and language scores (measured through the Bayley Scales of Infant and Toddler Development) compared to the control group [14]. The study also showed that mothers in the intervention group reported significantly lower depressive symptoms post-intervention [14]. This is relevant because maternal psychosocial problems can have an effect on neonatal outcomes, including cognitive development [17]. These findings provide evidence that interventions in early childhood to develop a nurturing household environment can attenuate the negative long-term effects of delayed cognitive development.

Determinants of developmental delay (e.g., maternal depression, lower socioeconomic status, and malnutrition) are more prevalent in LMICs than in high-income countries (HICs) [18, 19]. Despite the higher prevalence of these determinants in LMICs, the ramifications of some of these factors have not been well studied in LMIC settings and findings from HICs may not be generalizable to LMIC populations [18, 19]. Studies that have explored the determinants of early child development in LMICs have mostly focused on biological factors, enteropathogen infections [20], the validity of measuring scales [21], and child growth [22]. The limited research on the effects of non-biological determinants of child cognitive development has explored early infant cognitive outcomes at two or three years of age [3, 14, 23]. Trials investigating cognitive outcomes at later stages of childhood have focused on either fluid reasoning or verbal development and have used data from several different LMICs [24, 25]. Therefore, although studies have hypothesized long lasting effects of environmental and psychosocial factors on child outcomes, few have measured the site-specific impact of such factors at older ages [26].

This study investigates the effect of psychosocial and environmental determinants on child cognitive development at 5 years of age in rural South Africa and Tanzania. Understanding which geographically specific psychosocial and environmental factors have an impact on child cognitive development can inform further interventions aiming to alleviate the burden of compromised cognitive development for children in LMICs.

## Methods

### Study Design and Data

This analysis includes data from the Venda, South Africa and Haydom, Tanzania sites of the Etiology, Risk Factors, and Interactions of Enteric Infections and Malnutrition and the Consequences for Child Health and Development (MAL-ED) study [27–29]. This study was a multi-disciplinary prospective community-based birth cohort study in eight global sites (Bangladesh, Brazil, India, Nepal, Peru, Pakistan, South Africa, and Tanzania). From November 2009 to February 2017, mother and child dyads were enrolled shortly after birth and followed until 5 years of child age. The MAL-ED study design and description of the study sites has been extensively described elsewhere [27–30].

### Participants

A total of 576 pregnant women over a period of two years were enrolled in the South African (SA) and Tanzanian (TZ) sites. Each site was responsible for enrolling and following the cohort of children. Exclusion criteria were (1) family’s intention to move outside the area in the next 6 months, (2) mother’s age (< 16 years), (3) twin pregnancy, (4) underweight infant (< 1.5 kg), (5) presence of diagnosable congenital disease or severe neonatal disease, and (6) sibling’s enrollment in the study. For the present analysis, only children with cognitive development scores at 5 years of age were included in the analysis (N = 230 for SA; N = 171 for TZ).

### Data and definitions

The main outcome of interest was child cognitive development at 5 years (±30 days) of age. Cognitive development was assessed using the Wechsler Preschool Primary Scales of Intelligence (WPPSI). This clinical tool assesses cognitive function by testing children on six subscales (Block Design, Information, Matrix Reasoning, Picture Concepts, Word Reasoning, and Vocabulary). The WPPSI measures progress and functioning in areas such as problem-solving, thinking processes, and decision-making skills. Some items in the WPPSI were adapted to account for cultural differences and to reduce the potential for the test to be culturally bias (e.g., in the information subscale, shower was changed to bath or bucket) [31].

Because the WPPSI provides both subtest and composite scores, the outcomes of interest were treated as three continuous scores representing the children’s: (1) general cognitive development and functioning (Full Scale IQ), (2) verbal reasoning and comprehension and attention to verbal stimuli (Verbal IQ), and (3) fluid reasoning, spatial processing, and visual-motor integration (Performance IQ). In comparing these three outcomes, we assessed the role that psychosocial and environmental factors play not only on the overall child development but also in specific functioning domains (i.e., verbal and performance).

Maternal depression was assessed using the self-reporting questionnaire (SRQ-20) at 1, 6, 12, 24, 36, and 60 (±30 days) months of child age. The SRQ-20 consists of 20 dichotomously coded items. We used a reduced version of SRQ-20 (SRQ-16) for this analysis because it excludes items reflecting somatic symptoms and has been used previously in the MAL-ED cohort [32]. To distinguish between the effects of exposure to postpartum depression and prolonged exposure to depressive symptoms, we assessed (1) a measure of post-partum depressive symptoms defined by the average SRQ-16 scores at 1, 6, and 12 months of child age, (2) one measure of maternal depressive symptoms defined by the average SRQ-16 scores at 24 and 36 months of child age, and (3) one measure of maternal depressive symptoms defined by the SRQ-16 score at 60 months, or 5 years, of child age.

Socioeconomic status was assessed through the WAMI index (Water, Assets, Maternal Education and Income) [33]. This measure of household socioeconomic status includes: (1) access to improved water and sanitation, (2) wealth measured by ownership of a set of eight assets, (3) maternal education, and (4) monthly household income. This index has been standardized and validated across the eight MAL-ED study sites [33].

This study assessed environmental factors that may impact child development (i.e., organization of the environment, provision of play material, opportunities for stimulation, and cleanliness of the child) through the Home Observation for the Measurement of the Environment (HOME) tool [34]. This tool was also used to measure some psychosocial factors (i.e., responsivity of the caregiver, avoidance of restrictions and punishment, and promotion of child development). This assessment tool has been used in studies worldwide [35, 36]. Furthermore, it was adapted and validated across the eight international sites of the MAL-ED study [21]. The HOME variable was measured at 6, 24, and 36 (±15 days) months of child age. HOME assessments at each of the three points in time were averaged and coded dichotomously at the overall median (i.e., for both sites together). The organization of the environment (SA median [IQR]: 11.0 [10.3,11.5]; TZ median [IQR]: 4.3 [3.3, 5.5] and maternal education (SA median: [IQR]: 10.5 [9.0, 12.0]; TZ median [IQR]: 7.0, [3.0, 7.0] were coded dichotomously at the site-specific medians due to non-overlapping distributions of these variables across the two sites.

Following MAL-ED procedures, children were weighed and measured at enrollment. Weight at enrollment was converted to weight-for-age Z-scores (WAZ) following the WHO 2006 growth standards [37]. We used enrollment WAZ as a proxy for birthweight in the analysis because weight at birth was missing for some children and because age at enrollment varied from 0-17 days. Additionally, we conducted homogeneity tests to identify significant differences in associations between the two sites.

### Data analysis

We selected covariates based on a directed acyclic graph [38]. We used multivariable linear regression for the continuous WPPSI outcomes using SAS version 9.4. The model included (1) environmental factors (organization of the environment, provision of play material, opportunities for stimulation, cleanliness of the child, and WAMI index for socioeconomic status), (2) psychosocial factors (responsivity of the caregiver, avoidance of punishment, maternal depressive symptoms, and maternal education), (3) child birthweight, and (4) indicators for the fieldworker who collected the data on the home environment (HOME field assessors). We included the HOME field assessor as a covariate because the assessor was significantly associated with both the HOME inventory scale measurements and the WPSSI outcomes.

### Ethical approval

We obtained ethical approval from the Institutional Review Boards for the original and follow-up studies at the University of Venda (Limpopo, South Africa), at the Haydom Lutheran Hospital (Haydom, Tanzania), and the University of Virginia School of Medicine (Charlottesville, United States).

## Results

In this analysis, we included 401 (69.6%) children who had WPSSI scores at 5 years of age. Children were 50.6% female, had an average weight of 3.18 kg at birth, and 7.7% of them had a bodyweight of 2.50 kg or less at enrollment. Approximately 60% of mothers were married and 53.1% of them had fewer than 8.5 years of education. Almost a quarter of mothers presented with depressive symptoms postpartum (n= 91, 22.8%), at 24 and 36 months of child age (n=95, 24.8%), or at 60 months of child age (n=68, 17.3%) (Table 1). Women who presented depressive symptoms had relatively few symptoms, with only approximately 1% of them presenting 8 or more depressive symptoms on a 0-16 point scale. Children who did not have WPPSI measured and were not included in the analysis presented similar baseline characteristics. Baseline characteristics were also similar between the two sites with the exception of maternal education and opportunities for stimulation from the HOME index (Table 1).

**Table 1:** Characteristics of 401 mother child dyads characteristics in rural South Africa and Tanzania

Variables Count (%) or Median [IQR]	South Africa *n* = 230	Tanzania *n* = 171	Total *n* = 401	Missing *n* = 175
Sex	119 (51.7)	84 (49.1)	203 (50.6)	89 (50.9)
Bodyweight for Age	-0.32 [-0.94, 0.28]	-0.03 [-0.61, 0.58]	-0.18[-0.85, 0.38]	-0.36 [-0.94, 0.16]
Environmental Factors				
Organization of the Environment	132 (58.4)^*^	99 (57.9)	231 (58.2)^*^	64 (59.8)^*^
Provision of Play Material	128 (56.6)^*^	112 (65.5)	240 (60.5)^*^	69 (64.5)^*^
Opportunities for Stimulation	143 (63.3)^*^	139 (81.3)	282 (71.0)^*^	80 (74.8)^*^
Cleanliness of the Child	200 (88.5)^*^	64 (37.4)	264 (66.5)^*^	71 (66.4)^*^
WAMI – Socioeconomic Status	0.79 [0.71, 0.85]^*^	0.20 [0.13, 0.39]	0.66 [0.32, 0.80]^*^	0.30 (0.20, 0.51)^*^
Maternal Factors				
Responsivity of the Caregiver	68 (30.1)	136 (79.5)	204 (51.4)	76 (71.0)
Avoidance of Punishment	96 (42.8)	158 (92.4)	254 (64.0)	91 (85.1)
Promotion of Child Development	180 (79.7)	137 (80.1)	317 (79.9)	88 (82.2)
Depressive Symptoms 1,6, 12 mo.	53 (23.3)^*^	38 (22.2)	91 (22.8)^*^	43 (30.3)^*^
Depressive Symptoms 24, 36 mo.	62 (28.6)^*^	33 (19.9)^*^	95 (24.8)^*^	12 (30.8)^*^
Depressive Symptoms 60 mo.	46 (20.1)^*^	22 (13.4)^*^	68 (17.3)^*^	3 (17.7)^*^
Maternal Education > 7/10years	115 (50.0)	98 (57.3)	213 (53.1)	104 (59.4)

^*^ Missing

In the multivariable regression analysis including both sites, the WAMI index had the largest effect on cognitive development and was strongly associated with full scale IQ (Score Difference (SD):14.27, 95% CI:1.96, 26.59) and performance IQ. Opportunities for stimulation in the home environment were also associated with full scale IQ (SD: 3.18, 95% CI: 0.59, 5.76) and performance IQ. However, the WAMI index and opportunities for stimulation had smaller associations with verbal IQ in this cohort. The organization of the home environment was associated with full scale IQ (SD: 3.08, 95% CI: 0.65, 5.52) and was more associated with verbal IQ compared to performance IQ. Provision of appropriate play materials was associated with performance IQ. No maternal factors or other environmental factors measured by the HOME assessment in this study were associated with any of the three WPSSI outcomes (Table 2).

**Table 2:** Multivariable regression analysis of child development determinants in rural South Africa and Tanzania^*^

	Full Scale IQ SD^*^ [95% CI]	Verbal IQ SD^*^ [95% CI]	Performance IQ SD^*^ [95% CI]
**Environmental Factors**			
Organization of the Environment	3.08^*^[0.65, 5.52]	1.79^*^[0.32, 3.27]	1.29 [-0.06, 2.65]
Provision of Play Material	-0.66 [-3.01, 1.20]	0.76 [-0.66, 2.19]	-1.42^*^[-2.73, -0.11]
Opportunities for Stimulation	3.18^*^[0.59, 5.76]	1.42 [-0.15, 2.99]	1.76^*^[0.32, 3.20]
Cleanliness of the Child	1.20 [-1.77, 4.18]	0.42 [-1.38, 2.22]	0.78 [-0.87, 2.44]
Socioeconomic Status	14.27^*^[1.96, 26.59]	4.24 [-3.22, 11.71]	10.03^*^[3.17, 16.89]
**Maternal Factors**			
Responsivity of the Caregiver	-1.92 [-4.66, 0.81]	-1.18 [-2.83, 0.48]	-0.74 [-2.27, 0.78]
Avoidance of Punishment	1.58 [-1.38, 4.55]	0.42 [-1.38, 0.48]	1.17 [-0.48, 2.82]
Promotion of Child Development	-1.89 [-4.71, 0.93]	-0.81 [-2.51, 0.48]	1.09 [-2.66, 0.49]
Depressive Symptoms 1,6, 12 mo.	-0.31 [-3.03, 2.40]	0.72 [-0.93, 2.37]	-1.03 [-2.54, 0.48]
Depressive Symptoms 24, 36 mo.	1.40 [-1.26, 4.06]	1.53 [-0.09, 3.14]	-0.13 [-1.61, 1.35]
Depressive Symptoms 60 mo.	-1.27 [-4.33, 1.79]	-0.79 [-2.65, 1.06]	-0.47 [-2.18, 1.23]
Maternal Education > 7/10years	0.87 [-1.74, 3.49]	0.76 [-0.83, 2.34]	0.11 [-1.34, 1.57]

^*^ Estimates in the tables above are adjusted for site, birthweight, field assessors, and all other variables in the table.

^*^ SD: WPPSI Score Difference

^*^ P-value < 0.05

In the South African site, organization of the environment, opportunities for stimulation, and the WAMI index were associated with at least one WPSSI outcome. Similar to the analysis for both sites, the WAMI index had the strongest effects on cognitive development. Unique to South Africa, avoidance of punishment was associated with full scale IQ (SD: 4.05, 95% CI: 0.69, 7.42; p = 0.004 for homogeneity test to assess differences between sites) and performance IQ (SD: 2.31, 95% CI: 0.41, 4.20; p=0.006 for homogeneity test) (Table 3).

**Table 3:** Multivariable regression analysis of determinants for child development in rural South Africa*

	Full Scale IQ SD* [95% CI]	Verbal IQ SD* [95% CI]	Performance IQ SD* [95% CI]
**Environmental Factors**			
Organization of the Environment	3.62^*^[0.29, 6.95]	1.95 [-0.09, 3.99]	1.67 [-0.21, 3.54]
Provision of Play Material	1.42 [-1.82, 4.65]	1.79 [-0.19, 3.77]	-0.37 [-2.19, 1.45]
Opportunities for Stimulation	3.00 [-0.21, 6.22]	1.17 [-0.80, 3.13]	1.84^*^[0.03, 3.65]
Cleanliness of the Child	1.36 [-3.76, 6.49]	-0.27 [-3.41, 2.86]	1.63 [-1.25, 4.52]
Socioeconomic Status	16.00^*^[0.12, 31.89]	5.00 [-4.72, 14.72]	11.00^*^[2.06, 19.94]
**Maternal Factors**			
Responsivity of the Caregiver	-1.05 [-4.83, 2.74]	-0.60 [-2.92, 1.71]	-0.44 [-2.57, 1.69]
Avoidance of Punishment	4.05^*^[0.69, 7.42]	1.75 [-0.32, 3.81]	2.31^*^ [0.41, 4.20]
Promotion of Child Development	-1.29 [-5.05, 2.47]	-0.28 [-2.59, 2.01]	-1.00 [-3.13, 1.11]
Depressive Symptoms 1,6, 12 mo.	-2.83 [-6.46, 0.80]	0.79 [-3.00, 1.44]	-2.04^*^[-4.09, -0.00]
Depressive Symptoms 24, 36 mo.	2.73 [-0.65, 6.12]	2.21* [0.13, 4.28]	0.53 [-1.38, 2.44]
Depressive Symptoms 60 mo.	-1.17 [-5.20, 2.86]	-1.24 [-3.70, 1.22]	0.07 [-2.20, 1.23]
Maternal Education > 7/10years	1.02 [-2.29, 4.34]	0.86 [-1.17, 2.89]	0.16 [-1.71, 2,02]

^*^ Estimates in the tables above are adjusted for site, birthweight, field assessors, and all other variables in the table.

^*^ SD: WPPSI Score Difference

^*^ P-value < 0.05

In the Tanzania site, the provision of appropriate play material was inversely associated with full scale IQ (SD: -3.55, 95% CI: -6.91, -0.18; p=0.01 for homogeneity test) and performance IQ (SD: -2.75, 95% CI: -4.63, -0.87; p=0.03 for homogeneity test). Furthermore, the presence of depressive symptoms in the post-partum period (until one year after child birth) was associated with higher full scale IQ (SD: 3.93, 95% CI: 0.12, 7.74; p=0.03 for homogeneity test) and verbal IQ (SD: 3.11, 95% CI: 0.84, 5.39; p=0.02 for homogeneity test). Aside from these exceptions, other associations were consistent between sites (Table 4).

**Table 4:** Multivariable regression analysis of determinants for child development in rural Tanzania*

	Full Scale IQ SD^*^ [95% CI]	Verbal IQ SD^*^ [95% CI]	Performance IQ SD^*^ [95% CI]
**Environmental Factors**			
Organization of the Environment	1.29 [-2.07, 4.65]	1.29 [-0.71, 3.30]	-0.008 [-1.89, 1.87]
Provision of Play Material	-3.55^*^[-6.91, -0.18]	-0.80 [-2.80, 1.21]	-2.75* [-4.63, -0.87]
Opportunities for Stimulation	3.01 [-1.10, 7.12]	1.78 [-0.67, 4.23]	1.32 [-1.07, 3.53]
Cleanliness of the Child	1.45 [-2.07, 4.96]	0.61 [-1.48, 2.70]	0.84 [-1.12, 2.80]
Socioeconomic Status	13.30 [-1.53, 28.13]	3.93 [-4.91, 12.78]	9.37* [1.08, 17.66]
**Maternal Factors**			
Responsivity of the Caregiver	-2.37 [-6.37, 1.63]	-1.01 [-3.40, 1.37]	-1.35 [-3.59, 0.88]
Avoidance of Punishment	-5.99 [-12.55, 0.58]	1.75 [-0.32, 3.81]	-3.35 [-7.02, 0.32]
Promotion of Child Development	-1.81 [-5.78, 2.16]	-0.11 [-3.48, 1.26]	-0.70 [-2.92, 1.52]
Depressive Symptoms 1,6, 12 mo.	3.93^*^[0.12, 7.74]	3.11^*^[0.84, 5.39]	0.82 [-1.31, 2.95]
Depressive Symptoms 24, 36 mo.	-2.74 [-6.74, 1.26]	-0.92 [-3.31, 1.47]	-1.82 [-4.06, 0.42]
Depressive Symptoms 60 mo.	0.53 [-4.24, 5.30]	0.57 [-2.27, 3.42]	-0.04 [-2.70, 2.62]
Maternal Education > 7/10years	2.29 [-11.37, 15.96]	-6.02 [-14.16, 2.13]	8.31* [0.67, 15.84]

^*^ Estimates in the tables above are adjusted for site, birthweight, field assessors, and all other variables in the table.

^*^ SD: WPPSI Score Difference

^*^ P-value < 0.05

## Discussion

Socioeconomic status, the organization of the home environment, and opportunities for cognitive stimulation were associated with child cognitive development at 5 years of age among children in the South African and Tanzanian sites of the MAL-ED study. The strongest association with child cognitive development at 5 years of age was found for socioeconomic status (measured using the Water, Assets, Maternal Education and Income index). The WAMI index was previously shown to be associated with children’s cognitive development scores, measured with the Bayley Scale of Infant Development, at 15 months of age in the Tanzanian site of the MAL-ED cohort [23]. These results demonstrate not only the large effect of socioeconomic status on cognitive development but also its long-lasting impact up to 5 years of child age.

Intergovernmental organizations have recognized that poverty is related to suboptimal health and increased mortality [39]. However, there is less recognition of the role that poverty plays in children’s cognitive development in LMICs due to the lack of national statistics on children’s cognitive development. This study’s finding contributes to the growing body of literature showing the association between socioeconomic status and children’s cognitive development [3, 40]. Reducing income inequalities and increasing opportunities for social mobility in LMICs can help diminish the economic divide between HICs and LMICs and contribute to the goal of achieving global health equity.

This study also found a modest association of the organization of the home environment and opportunities for cognitive stimulation on child cognitive development at 5 years of age. The results of these analyses show that an appropriate home environment (e.g., with clean, organized, hazard-free areas for children to play) where caregivers provide adequate stimulation (e.g., promoting recreational and learning materials and activities) may positively impact children’s cognitive development. In this context, we also found opportunities for stimulation and learning (e.g., presence of toys, books, and interactions with relatives) to be positively associated with children’s cognitive development.

Consistent with this perspective, other studies have found cognitive stimulation to be associated with children’s cognitive ability and academic achievement [40]. For example, Cooper et al. randomized women in South Africa to an intervention aimed to educate mothers about sensitive and responsive parenting. The researchers found that the intervention had a significant impact on mother-child relationships and predicted child development. The role that cognitively stimulating materials and experiences play in cognitive development is not only recognized in the academic literature but is also well established in global policy practices. In the 2007 *Lancet* series on Child Development in Developing Countries, the International Child Development Steering Group (ICDSG) identified factors with sufficient evidence to recommend implementing prevention strategies [2, 40]. These factors include inadequate provision of cognitively stimulating materials, growth retardation and low birth weight, and illnesses.

### Limitations

Because these data were from two specific rural sites in South Africa and Tanzania, the generalizability of the present findings may be limited. Future research should replicate and expand this work in rural and urban settings as well as in other LMICs. The findings of this study are also limited by the age of the children in the cohort as cognitive development was assessed at 5 years of age, when cognitive abilities may not have stabilized. Future research should investigate the role that psychosocial and environmental factors play at other stages of child development.

In this study, cognitive development was assessed using the WPPSI-III assessment tool, which assesses cognitive function by testing children on eight subscales. However, the adapted assessments did not include the word reasoning score. This limits the generalizability of these results to other assessments using the WPPSI and may have had an impact in the estimates presented in this analysis as we were not able to capture score differences within this domain. We adjusted for field assessors in the analysis to account for potential confounding by field assessor, but the limited associations found between the HOME index and the WPSSI may be in part due to measurement error.

In this analysis, maternal depressive symptoms were not significantly associated with any of the WPSSI outcomes. These findings are in contrast with past studies in LMICs that have shown that suboptimal maternal mental health is associated with poor child growth and development [41]. The lack of significant association can potentially be attributed to the use of the self-reported questionnaire to assess maternal mental health and the relatively low frequency of reports of depressive symptoms among women in this cohort, which could have underpowered the study to detect this relationship. Similarly, site-specific inconsistent effects on child cognitive development may in part reflect cultural differences in how women report depressive symptoms and the difficulty of assessing child development across diverse low-resource settings.

## Conclusion

This study shows a stronger association with child cognitive development at 5 years of age for socioeconomic status compared to other psychosocial and environmental factors which, we had hypothesized, were more proxy determinants of child cognitive development. This demonstrates the large and long-lasting effect that socioeconomic status has on child cognitive development which contributes to the economic and health divide between HICs and LMICs. Because the limited associations between the HOME index and the WPSSI may be in part due to difficulty in measuring these constructs in low-resource settings, future studies should further investigate measures of psychosocial and environmental factors that may affect child cognitive development. A more comprehensive understanding of the context in which children grow and develop cognitively is necessary to inform interventions aiming to alleviate the burden of compromised cognitive development for children in LMICs.

## Data Availability

The MAL-ED dataset supporting the conclusion of this article can be accessed publicly through the ClinEpiDB platform (at ClinEpiDB.org).

## Abbreviations

HIC(s): High-income Country (ies)
LMIC(s): Low- and Middle-income Country (ies)
MAL-ED: Etiology, Risk Factors, and Interactions of Enteric Infections and Malnutrition and the Consequences for Child Health and Development
WPPSI: Wechsler Preschool Primary Scales of Intelligence
SRQ-20: Self-reporting Questionnaire
WAMI: Water, Assets, Maternal Education and Income Index
HOME: Home Observation for the Measurement of the Environment
